# Influence of exercise prescription intervention based on WeChat on glycolipid metabolism and fitness of suboptimal-health teachers

**DOI:** 10.1097/MD.0000000000038167

**Published:** 2024-05-24

**Authors:** Yimei Duan, Shunchang Li, Quansheng Su, Simao Xu, Guotian Lu

**Affiliations:** a Institute of Sports Medicine and Health, Chengdu Sports University, Chengdu, Sichuan, China; b College of Physical Education, Sichuan Normal University, Chengdu, Sichuan, China; c College of Physical Education, Guangxi Normal University, Guilin, Guangxi, China.

**Keywords:** exercise intervention, glycolipid metabolism, physical fitness, suboptimal-health status, WeChat

## Abstract

Exercise is an effective means to promote health, but adherence is low. Due to the advantages of immediacy, economy and effectiveness, the use of WeChat social software has permeated into every aspect in daily life in China. To explore the influence of WeChat-based exercise prescription intervention mode on glycolipid metabolism and fitness of suboptimal-health teachers. 293 suboptimal-health teachers with senior professional titles were randomized to a control group (CG) or an experimental group (e.g.). The CG exercised on its own, while the e.g. adopted the exercise prescription intervention based on WeChat. The intervention period was 6 months. Finally, 264 cases were adhered to and completed, including 132 cases in the CG and 132 cases in the e.g.. The Suboptimal-Health Status Questionnaires-25 scores (SHSQ-25 scores), exercise adherence, subjective feelings, physical fitness, blood glucose and blood lipids were detected before and after intervention and compared between 2 groups. After the intervention, the SHSQ-25 scores in the e.g. was significantly decreased than those in the CG (*P* < .01). The complete exercise adherence in the e.g. was significantly higher than those in the CG (*P* < .01). After intervention, the subjective feelings of e.g. were significantly improved compared to CG (*P* < .05). The body shape, body function and physical quality in the e.g. was higher than those in the CG (*P* < .05). Total cholesterol (TC), triglyceride (TG), low-density lipoprotein cholesterol (LDL-C) decreased significantly in the e.g. but not in the CG (*P* < .05). Fasting blood glucose (FBG) decreased significantly in the e.g. but not in the CG, with a significant difference between groups (*P* < .05). The subjects in the e.g. were very satisfied with WeChat management. WeChat-based exercise prescription intervention could improve SHS, exercise adherence, subjective feelings, physical fitness and glycolipid metabolism.

## 1. Introduction

In 1989, N. Buchman, a scholar from the former Soviet Union, first proposed that there was a third state between health and disease, also known as “pre-illness state,” which was collectively called “chronic fatigue syndrome,” which was translated as “suboptimal-health status (SHS)” by Chinese scholars. Appropriate exercise and reasonable diet are 2 important factors to ensure health, and are also effective ways to prevent and treat chronic diseases at all levels. Unhealthy lifestyle is significantly related to the risk of SHS.^[1]^ Suboptimal-health state refers to a kind of low-quality health condition between health and disease, such as lack of energy, memory loss, inattention, worrying about their health, dreaminess, worry, irritability, emotional instability, feeling very tired, poor response, drowsiness and reduced adaptability.^[2]^ The main symptoms of SHS are “three highs”: hyperlipemia, hyperglycemia and hypertension close to the critical level. According to the Statistical Bulletin on the Development of China Health Care in 2019, the average life expectancy of Chinese residents has increased to 77.3 years. However, it is reported that the average life expectancy of Chinese intellectuals is much lower than the average.^[3]^

A survey of teachers’ living conditions shows that more than 80% of teachers express great stress. Epidemiological investigation shows that teachers are a high-risk occupation of suboptimal-health, and 40 to 50 years old is a high-risk age group.^[4]^ Chinese university teacher takes on the tasks of teaching, scientific research and management, and stress increases sharply due to factors such as professional title evaluation, education promotion, salary and welfare. Meanwhile, the serious lack of physical activity leads to an increase in the incidence of suboptimal-health, the phenomenon of overwork and premature death has been reported from time to time.^[2]^ Steven Blair,^[5]^ a famous American sports epidemiologist, pointed out that lack of sports and physical activity would become the biggest public health problem in the 21st century. Insufficient physical activity was significantly correlated with suboptimal-health. Exercise prescription is an exercise program that determines its exercise items, exercise forms, exercise intensity, exercise time, exercise frequency and precautions in the form of prescription.

Teachers with senior titles are the core of university and family. The SHS is bidirectional, which may develop into a disease state or reverse into a healthy state depending on the subjects’ attitude toward sedentarism. SHS is associated with chronic diseases such as diabetes and cardiovascular disease.^[6,7]^ Accumulating evidences suggests that physical exercise is a protective factor for SHS and psychological symptoms,^[8–10]^ but exercise adherence is low.^[11,12]^ With the popularity of smartphones and the high audience of WeChat software, WeChat has become the largest APP in China. According to the 2023 WeChat Annual Data Report, the total number of WeChat users has reached 1.2 billion. WeChat platform has the characteristics of immediacy, convenience, economy, effectiveness and safety, making WeChat guidance an effective supplement to health intervention forms.^[13,14]^ This study investigated the effects of WeChat-based exercise intervention on exercise adherence, subjective feelings, physical fitness and glycolipid metabolism through the personalized exercise prescription, with the aim of establishing a sense of health management, and shifting from “treating disease” to “preventing disease.”

## 2. Materials and methods

### 2.1. Research object

Inclusion criteria: Professional title: senior professional title, Individuals with no major physical and mental diseases based on clinical diagnosis, Suboptimal-Health Status Questionnaires-25 (SHSQ-25) sore ≥ 35. Exclusion criteria: non-senior title, Retired or no longer at work, (Unwilling to participate in the survey. According to the inclusion criteria and exclusion criteria, 295 subjects aged 32 to 60 years old were obtained.

In this study, SHSQ-25 was used to measure the SHS. The Cronbach α coefficient of this scale was 0.91, which was proved to have good reliability and validity.^[15]^ SHSQ-25 contains a total of 25 items in 5 domains: fatigue, mental status, immune system, gastrointestinal tract, and cardiovascular system.^[16]^ SHSQ-25 has been proven to be an effective health assessment tool for ethnically diverse health surveys.^[8,17,18]^ SHSQ-25 was developed in China, then adapted to the Chinese language and population.^[16,19]^ Individuals are scored according to the frequency of experiencing symptoms in the past 3 months, and the addition of 25 entries’ scores greater than or equal to 35 (SHSQ-25 scores ≥ 35) is considered to be SHS.^[20]^

The research objects were selected from Dali University, Sichuan Normal University and other 10 universities. 295 subjects were divided into an experimental group (e.g., 148 cases) and control group (CG, 147 cases) by random number table method, and a total of 264 cases were adhered to and completed in the end, including 132 cases in the CG and 132 cases in the e.g. CG: 31 females and 101 males, with an average age of (44.16 ± 10.52) years. e.g.: 32 females and 100 males, with an average age of (44.78 ± 10.31) years. All subjects had been informed of the purpose and process of the study and had signed informed consent. This study had been approved by the Ethics Committee (2021–2027) in accordance with the Helsinki Declaration. The basic information of the 2 groups is shown in Table 1. According to statistical analysis, the baseline characteristics were balanced.

**Table 1:** Comparison of general data (n = 264).

Parameter	CG (n = 132)	e.g. (n = 132)	*t/χ*^2^ value	*P* value
Age (yr)	44.16 ± 10.52	44.78 ± 10.31	0.484	.629
Height (cm)	167.93 ± 13.47	168.45 ± 12.35	0.327	.734
Weight (kg)	64.46 ± 7.31	65.37 ± 8.02	0.963	.336
Physical inactivity	72 (54.55%)	75 (56.82%)	0.138	.804
SHSQ-25 scores	35.6 ± 2.6	35.3 ± 2.1	0.539	.592

Before intervention, the physical activity of the 2 groups of subjects was investigated by questionnaire. Physical inactivity was defined as exercising <3 times a wk, exercising for <10 min each time, and exercising with a heart rate of <110 beats/min.

CG = control group, e.g. = experimental group.

### 2.2. Research methods

#### 2.2.1. Intervention methods

##### 2.2.1.1. CG intervention method

On the basis of the original lifestyle, the CG was verbally reminded of the importance of exercise regularly (once a month), but did not provide detailed guidance. The duration of intervention was 6 months.

##### 2.2.1.2. E.g. intervention method

The e.g. formed a “suboptimal-health intervention WeChat group,” and the research team members and experimental subjects joined the group. The research team served as the guidance work, including sports prescribers, sports rehabilitation therapists, fitness coaches, physical training experts, social sports instructors, etc. WeChat group regularly pushes 4 to 6 articles and public accounts on suboptimal-health knowledge, exercise prescription, health education and health management knowledge every week to guide patients to exercise scientifically. Filled in the exercise diary: The subjects were reminded to fill in the exercise diary and given guidance on the day before each week. Uploaded fitness videos: Subjects were required to upload fitness videos once every 2 weeks, the instructors would give suggestions based on the videos. Held WeChat group lectures: Held a health education lecture every 2 weeks, the content of the lecture was focused on “sports, suboptimal-health and disease,” 40 minutes/time. Held experience-sharing meeting: Selected subjects with good execution ability every month and shared personal experience in WeChat group, 30 to 60 minutes each time. Consulted at any time and replied in time: Subjects were encouraged to consult through WeChat group at any time when they had questions, and the instructors would reply in time. Implemented of humanistic care: instructors used WeChat group to communicate with subjects regularly, paid attention to mental health.

Exercise intervention was as follows: exercise 5 times a week, 60 minutes each time, divided into 3 parts (preparation activity 10 minutes, formal activity 40 minutes and finishing activity 10 minutes). The exercise prescription included aerobic exercise, resistance exercise and flexibility exercise, and the intervention period was 6 months. Recommend walking/running, aerobics, square dancing, traditional Chinese health exercises, ball games, swimming and other aerobic exercises, as well as resistance exercises such as elastic bands, elastic ropes, dumbbells and barbells. The subjects chose according to their interests. The heart rate index combined with the subjective Sensory Assessment Exercise Scale (RPE) to evaluate the exercise intensity. Fitbit Alta was used to monitor heart rate. Before the intervention, 1RM (one-repetition maximum) of the experimental subjects were calculated. The subjects tried to find a charge they were able to repeat 10 times with a sensation of weakness at the end. On the basis of this information, they made an estimative of 1RM to choose a training weight or elastic resistance that provided around 60% to 70% of the 1 RM to be in the American College of Sports Medicine.^[21]^ Using elastic bands, elastic ropes, dumbbells and barbells, the training load was 60% to 70% 1RM, 8 to 12 times/group, 3 groups.^[22]^ Flexibility exercises were performed through stretching exercises and proprioceptive neuromuscular promotion therapy (PNF). 10 to 15 repetitions for each movement, 2 sets in total, 3 minutes rest between sets.

#### 2.2.2. Index selection and testing

Step test, 1-minute sit-up, pushup, sit and reach were measured by the Korean HELMAS II exercise fitness assessment system. Before and after the intervention, subjects in both groups completed the following indicators tests in the human kinesiology laboratory and affiliated hospitals. Step test, 1-minute sit-up, pushup, sit and reach were measured by the Korean HELMAS II exercise fitness assessment system. The step test involves subjects completing an up-and-down stair climbing exercise at a frequency of 30 repetitions/minutes for 3 minutes. Immediately after the exercise, the 3 pulse counts were measured from 1 minute to 1 minute 30 seconds, 2 minutes to 2 minutes 30 seconds, and 3 minutes to 3 minutes 30 seconds after the exercise. The recorded results were then used to calculate the step index to determine the function of the cardiovascular system. The 1-minute sit-up requires the subject to lie on their back on a mat with their legs slightly apart and their knees bent at about a 90-degree angle. Subjects sat up with both elbows touching both knees as completion of 1 rep, and the number of reps completed in 1 minute was recorded. pushup was performed by having the subject brace his/her hands on the ground, with the hands spaced shoulder-width apart, keeping the body straight, bending the arms to bring the body down flat to the point where the shoulders and elbows were at the same level, and then bracing the body up to record the number of times this was accomplished. The sit and reach were performed by having the subject legs straight, heels together, and upper body bent forward. Used the fingertips in both hands to gradually push forward the cursor of the sit-and-reach tester until it cannot be pushed forward, and recorded the distance of the cursor forward. The OMRON HEM-6050 electronic sphygmomanometer detected blood pressure (SBP/DBP). The Korean Biospace Inbody720 body composition analyzer tested body fat percentage. Hitachi 7600 automatic biochemical analyzer to detect fasting blood glucose (FBG), total cholesterol (TC), triglyceride (TG), high-density lipoprotein cholesterol (HDL-C), low-density lipoprotein cholesterol (LDL-C). Referring to the evaluation criteria of exercise adherence,^[23]^ fill in the exercise record form for assessment, and compare the proportion of complete adherence, partial adherence, and non-adherence between the 2 groups. Complete adherence meant achieving 80% or more of the recommended total exercise time per week; Partial adherence meant reaching 50% to 80% of the recommended total exercise time per week; Non-adherence was defined as <50% of the recommended total exercise time per week. Referring to the study of Stefan RR et al,^[24]^ the Subjective Feelings Evaluation Questionnaire was designed to evaluate the subjective feelings of the 2 groups, which included good mood, attention concentration, emotional stability, full of energy, increased interest, sufficient physical strength, improved sleep, improved diet and stress relief. The reliability and validity of the questionnaire were good after pretest. The changes of physical fitness and glycolipid metabolism were reflected by body shape, body function, physical quality, blood lipid and blood sugar indexes.

From the first month to the sixth month, the 2 groups conducted monthly telephone and WeChat group follow-up. After 6 months of intervention, the indexes of both groups before and after intervention were detected.

### 2.3. Statistical methods

Epidata 3.1 software and SPSS20.0 statistical software were used for data processing. The measurement data were expressed by Mean ± SD, the comparison was made by t test or covariance analysis. Counting data was expressed in rate (%), and *χ*^2^ test was used for comparison. Grade data were expressed by frequency, and rank sum test was used for comparison, with significant level α = 0.05.

## 3. Result

### 3.1. Comparison of SHSQ-25

The comparison of SHSQ-25 scores is shown in Table 2. There was a significant decrease in SHSQ-25 scores in the e.g. compared to the pre-intervention period *(P* < .01), whereas there was no significant change in the CG after the intervention (*P* > .05). In addition, SHSQ-25 scores decreased significantly in the e.g. after the intervention compared to the CG after the intervention (*P* < .01).

**Table 2:** Comparison of SHSQ-25 [n = 264, (Mean ± SD)].

Parameter	CG (n = 132)		e.g. (n = 132)	
	Pre-intervention	Post-intervention	Pre-intervention	Post-intervention
SHSQ-25 scores	35.6 ± 2.6	33.0 ± 1.8	35.3 ± 2.1	28.1 ± 1.3^*◆^

CG = control group, e.g. = experimental group.

*P < .05 vs pre-intervention (within the group), ^◆^*P* < .05 vs pre-intervention (CG).

### 3.2. Comparison of exercise adherence

In the CG, the proportions of complete adherence, partial adherence and non-adherence were 28.03%, 46.97%, and 25.00%. In the e.g., the proportion of complete adherence, partial adherence and non-adherence were 58.33%, 25.76%, and 15.91%. After rank sum test, the difference between 2 groups was statistically significant (Z = −4.246, *P* = .000), and the adherence of the e.g. was higher than CG (Table 3).

**Table 3:** Comparison of exercise adherence [n = 264, case (%)].

Adherence	Complete adherence	Partial adherence	Non- adherence
CG	37 (28.03%)	62 (46.97%)	33 (25.00%)
e.g.	77 (58.33%)	34 (25.76%)	21 (15.91%)

CG = control group, e.g. = experimental group.

### 3.3. Comparison of subjective feelings

The comparison of subjective feelings was shown in Table 4. Compared to the CG, the e.g. showed improvement in mood, concentration, emotional stability, energy, interest, physical strength, sleep improvement, diet improvement and stress relief, and the difference was statistically significant (*P* < .05).

**Table 4:** Comparison of subjective feelings [n = 264, case (%)].

Subjective feeling	CG (n = 132)	e.g. (n = 132)	*χ* ^2^	*P*
Be in a good mood	78 (59.09%)	97 (73.48%)	6.119	.019
Attention concentration	53 (40.15%)	72 (54.55%)	5.485	.026
Emotional stability	82 (62.12%)	102 (77.27%)	7.174	.011
Full of energy	51 (38.64%)	83 (62.88%)	15.519	.000
Increased interest	32 (24.24%)	49 (37.12%)	5.147	.032
Physical strength	45 (34.09%)	68 (51.52%)	8.185	.006
Sleep improvement	28 (21.21%)	47 (35.61%)	6.723	.014
Diet improvement	37 (28.03%)	55 (41.67%)	5.405	.028
Stress relief	25 (18.94%)	44 (33.33%)	7.083	.011

CG = control group, e.g. = experimental group.

### 3.4. Comparison of physical fitness indexes

The comparison of physical fitness indexes was shown in Table 5. The body fat rate, systolic blood pressure, diastolic blood pressure, step test, 1 minute sit-up, pushup, sit and reach of the e.g. after intervention were significantly different from those before intervention (*P* < .05). There was no significant change in the CG before and after intervention. After intervention, the above indexes in the e.g. were significantly different from those in the CG (*P* < .05).

**Table 5:** Comparison of physical fitness indexes [n = 264, (Mean ± SD)].

Parameter		CG (n = 132)		e.g. (n = 132)	
		Pre-intervention	Post-intervention	Pre-intervention	Post-intervention
Body shape	Body fat rate (%)	25.68 ± 6.81	25.51 ± 5.14	25.74 ± 6.19	23.12 ± 3.63*^,◆^
Body function	Systolic blood pressure (mm Hg)	125.54 ± 9.32	126.03 ± 7.67	125.13 ± 9.19	116.76 ± 7.07^**◆◆^
	Diastolic pressure (mm Hg)	82.10 ± 6.57	81.92 ± 5.43	82.71 ± 6.36	80.04 ± 6.78
	Step test	48.17 ± 4.12	48.68 ± 3.33	48.47 ± 4.30	55.29 ± 3.36^**◆◆^
Physical quality	1 min sit-up (female) (times)	7.20 ± 2.76	7.65 ± 2.31	7.59 ± 3.21	10.29 ± 3.19^**◆◆^
	Pushup (male) (times)	12.51 ± 4.32	12.47 ± 3.31	12.71 ± 5.49	15.43 ± 3.52^**◆◆^
	Sit and reach (cm)	8.23 ± 3.25	8.36 ± 2.79	8.40 ± 4.01	11.16 ± 2.97**^◆◆^

CG = control group, e.g. = experimental group.

* *P* < .05 vs pre-intervention (within the group), ^◆^*P* < .05 vs pre-intervention (CG), **P < .01 vs pre-intervention (within the group), ◆◆*P* < .01 vs pre-intervention (CG).

### 3.5. Comparison of glycolipid metabolism indexes

After intervention, compared to the CG, TC, TG, LDL-C and FBG of e.g. were decreased, and the difference between 2 groups was statistically significant (*P* < .05). There was a significant difference between the above indexes before and after intervention in the e.g. (*P* < .05). However, the CG did not change significantly before and after intervention. The difference in high-density lipoprotein cholesterol (HDL-C) levels between 2 groups was not statistically significant, which may be explained by the short intervention time and the small sample size (Table 6).

**Table 6:** Comparison of glycolipid metabolism [n = 264, (Mean ± SD)].

Parameter	CG (n = 132)		e.g. (n = 132)	
	Pre-intervention	Post-intervention	Pre-intervention	Post-intervention
TC (mmol/L)	5.15 ± 0.54	5.12 ± 0.43	5.14 ± 0.61	4.83 ± 0.33*,◆
TG (mmol/L)	1.58 ± 0.54	1.57 ± 0.33	1.61 ± 0.49	1.38 ± 0.31*,◆
HDL-C (mmol/L)	1.43 ± 0.19	1.46 ± 0.23	1.44 ± 0.20	1.48 ± 0.28
LDL-C (mmol/L)	3.13 ± 0.47	3.09 ± 0.34	3.12 ± 0.42	2.88 ± 0.33*,◆
FBG (mmol/L)	5.76 ± 0.37	5.73 ± 0.28	5.80 ± 0.46	5.47 ± 0.32*,◆

CG = control group, e.g. = experimental group, FBG = fasting blood glucose, HDL-C = high-density lipoprotein cholesterol, LDL-C = low-density lipoprotein cholesterol.

* *P* < .05 vs pre-intervention (within the group),

◆ *P* < .05 vs pre-intervention (CG).

## 4. Discussion

The factors that affected the suboptimal-health teachers in Chinese universities included high ideological pressure, irregular life schedule, poor sleep quality, unhealthy lifestyle, long screen time, and improper fitness and exercise methods and so on. In view of the above situation, this study specially designed individualized exercise prescription and analyzed its effect. The results showed that the exercise prescription intervention based on WeChat could significantly reduce the score of SHSQ-25, and had a positive effect on improving suboptimal-health status.

The results of this study showed that the proportion of complete adherence and partial adherence in the CG was 28.03% and 46.97%, which was significantly lower than the proportion of 58.33 % and 25.76 % in the e.g.. This showed that the adherence of exercise intervention was good. Exercise intervention could improve the adherence of disease treatment,^[25]^ which laid a good foundation for further exercise. The literature showed that 1-year strength training with good adherence could improve the physiological parameters such as muscle strength, muscle mass and abdominal fat of healthy and chronically ill elderly people.^[26]^ The effect of this study combined with WeChat to improve exercise adherence was significant, which was consistent with previous studies.^[27–30]^ The possible reasons were as follows: First, the characteristics of WeChat were used. The WeChat platform integrated text, pictures, audio, video and other information transmission methods, which was easy to stimulate learning interest. Moreover, the senior titles teachers had a certain degree of education, could skillfully operate WeChat software, which was beneficial to improve adherence. Second, health education had been strengthened. Pushed knowledge through WeChat platform and communicated with subjects at any time to solve problems encountered in sports. Various forms of lectures and exchange sessions were held to enhance their drive and exercise awareness. Third, it enhanced self-management.^[14,30,31]^ Participants were encouraged to upload exercise videos through WeChat groups, clock in regularly, publish WeChat moments, record exercise diaries, etc, to strengthen self-supervision and self-efficiency. Fourth, played the role of peer model demonstration to encourage each other and improve positive energy. Fifth, reminding and urging through WeChat group was simple and convenient, which was conducive to control and easy for subjects to accept, and was an important driver to improve adherence. Sixth, promoted health education through the creation of easy-to-understand and popular exercise mantra - “aerobic every day, strength every other day, flexibility at any time” and other slogans.

In this study, compared to the CG, after the exercise prescription intervention, the e.g. had improved mood, attention, emotional stability, energy, interest, physical strength, sleep improvement, diet improvement and stress relief. Dragana et al^[32]^ found that the development of physical activities could reduce pain and improve subjective feelings. A certain intensity of water walking training could improve the subjective feelings of middle-aged and elderly women, reduce heart rate, improve metabolism, so as to improve physical fitness.^[33]^ A study found that moderate physical activity can counteract mental fatigue.^[34]^ Studies had shown that exercise interventions played a positive role in physical function, mental health, diet, sleep, brain and cognitive outcomes.^[35,36]^ In conclusion, exercise had a positive effect on reducing musculoskeletal pain, neurological headaches, sleep disorders, mental stress, oxidative stress, depression, anxiety, maintaining a pleasant mood, and enhancing the diversity of human intestinal flora.^[37–44]^ Therefore, it was recommended that the role of “Exercise is medicine” be effectively utilized through scientific exercise prescription.^[45–48]^

In order to further objectively analyzed the effect of exercise intervention, the e.g. was higher than the CG in the improvement of aerobic work capacity, muscle strength, muscle endurance and flexibility in terms of physical indexes. Compared to the CG, the e.g. improved in body fat, blood lipids, blood sugar, systolic blood pressure and diastolic blood pressure, which suggested that exercise intervention had positive effects on body shape, body function, body fitness and glucolipid metabolism. Exercise interventions could improve frailty by enhancing muscle strength and physical function in frail elderly people.^[49]^ It had been found that exercise could reduce blood viscosity, control blood pressure,^[50]^ improve the ability of skeletal muscle to take up and use oxygen, enhance myocardial contractility,^[51]^ improve autonomic nervous system and regulate emotions.^[52]^ Aerobic exercise combined with resistance training could significantly improve glucose and lipid metabolism in patients with type 2 diabetes and reduce inflammation.^[53]^ Both aerobic exercise and high-intensity interval training could significantly improve the body shape of obese male college students.^[54]^ A review suggested that appropriate exercise was effective in improving health-related parameters in patients with a variety of clinical conditions and diseases (e.g., chronic fatigue syndrome, Parkinson disease, psychological disorders, chronic neck pain, and chronic obstructive pulmonary disease).^[55]^ A study found that exercise frequency was significantly associated with SHS and psychological symptoms. Improvements were most pronounced when exercising 3 or more times per week.^[8]^ Other study had shown that Baduanjin exercise for 12 weeks could adjust the imbalance of traditional Chinese medicine physique in suboptimal-health patients. This study was consistent with previous studies.^[9]^ Exercise was a cornerstone of preventive medicine and a promising form of intervention.

From the first month to the sixth month, the 2 groups carried out monthly telephone follow-up and WeChat group follow-up. Through follow-up, it was found that the e.g. was satisfied with the exercise prescription intervention mode based on WeChat. For example, Wang gained a sense of accomplishment by receiving praise from her WeChat groupmates, Zhang gained a sense of satisfaction by meeting like-minded friends in the WeChat group, and the sharing and communication in the WeChat group made everyone feel more comfortable. Studies have found that exercise could promote the release of endorphins, dopamine, 5-hydroxytryptamine, norepinephrine and other happy hormones, resulting in a sense of pleasure, satisfaction, comfort and smooth experience, and played a positive intervention role,^[56]^ which may be the improvement mechanism. Exercise reduced depression levels by reducing inflammation, moderate-intensity exercise had the best effect.^[57]^ In this study, the exercise intervention based on WeChat broke through the limitation of time and space, so that the subjects learnt and communicated anytime and anywhere. Relevant content could be retained in the WeChat group for a long time, which was convenient for playback and review, and helped to improve subjects’ satisfaction. Through the humanistic care of WeChat group, the subjects’ trust and dependence on the instructors were enhanced, which was beneficial to the improvement of satisfaction.

However, there were some limitations in the study. This experiment did not conduct a comparative analysis of different genders and different ages, and further in-depth and detailed studies were needed.

In summary, the combination of health education and exercise intervention methods using WeChat as a medium could significantly improve suboptimal-health teachers’ exercise adherence and satisfaction, and improve their subjective feelings, exercise ability and medical-related indicators. However, the intervention information should be detailed and accurate, the intervention method should be targeted, the interaction, feedback and tracking with the subjects should be strengthened.

## Acknowledgments

We thank all participants for their hard work.

## Author contributions

**Conceptualization:** Yimei Duan.

**Formal analysis:** Shunchang Li.

**Funding acquisition:** Simao Xu.

**Investigation:** Shunchang Li, Quansheng Su.

**Methodology:** Quansheng Su.

**Software:** Simao Xu, Guotian Lu.

**Writing – original draft:** Yimei Duan.

**Writing – review & editing:** Guotian Lu.
